# Late recurrent gastric carcinoma 12 years after surgery with attenuation of CD44 variant 9 expression

**DOI:** 10.1186/s40792-023-01660-5

**Published:** 2023-05-22

**Authors:** Hirokatsu Hayashi, Itaru Yasufuku, Toshiya Higashi, Wakana Chikaishi, Ryoma Yokoi, Masahiro Fukada, Yuta Sato, Ryuichi Asai, Jesse Yu Tajima, Chiemi Saigo, Akitaka Makiyama, Yoshihiro Tanaka, Naoki Okumura, Katsutoshi Murase, Takao Takahashi, Manabu Futamura, Tamotsu Takeuchi, Nobuhisa Matsuhashi

**Affiliations:** 1 Department of Gastroenterological Surgery and Pediatric Surgery, Gifu University Graduate School of Medicine, 1-1 Yanagido, Gifu City, 501-1194 Japan; 2 United Graduate School of Drug Discovery and Medical Information Sciences, Gifu University, 1-1 Yanagido, Gifu City, 501-1194 Japan; 3 Cancer Center, Gifu University Hospital, 1-1 Yanagido, Gifu City, 501-1194 Japan; 4 Department of Breast Surgery, Gifu University Graduate School of Medicine, 1-1 Yanagido, Gifu City, 501-1194 Japan; 5 Department of Pathology and Translational Research, Gifu University Graduate School of Medicine, 1-1 Yanagido, Gifu City, 501-1194 Japan

**Keywords:** CD44 variant 9, Gastric cancer, Late tumor recurrence

## Abstract

**Background:**

Late recurrence of gastric cancer at 10 years post-gastrectomy is extremely rare, and the underlying mechanism remains unclear. We report a para-aortic lymph node metastasis case that recurred 12 years postoperatively.

**Case presentation:**

A 44-year-old woman pathologically diagnosed with moderately to poorly differentiated adenocarcinoma with pT2(SS)pN2cM0pStageIIIA according to the Japanese Classification of Gastric Carcinoma (the 13th Edition) underwent laparoscopic distal gastrectomy with D1 + lymph node dissection. She received adjuvant chemotherapy with tegafur-uracil (400 mg/day) for 2 years. At postoperative year (POY) 5, a swollen lymph node was detected in the No.16b1lat lymph node station. However, positron emission tomography (PET) revealed normal uptake, and the levels of tumor markers were within normal limits; hence, the possibility of metastasis was considered low, and the patient was placed under observation. At POY 12, computed tomography revealed an enlargement of the No.16b1lat lymph node station, and PET showed abnormal uptake. Endoscopic ultrasound-guided fine-needle aspiration revealed a moderately differentiated adenocarcinoma. Hence, a diagnosis of recurrence of gastric cancer was made. The patient underwent para-aortic nodal dissection (PAND) of No.16b1lat & int stations. Immunochemical staining results also suggested the recurrence of gastric cancer. However, the expression of CD44 variant 9 (CD44v9), a cancer stem cell marker for gastric adenocarcinoma, was attenuated in the recurrent lesions compared with that in the primary lesions. Postoperatively, she received chemotherapy with tegafur–gimeracil–oteracil (80 mg/day) for 1 year. Bone metastasis was observed at POY 4 after PAND, and the IHC analysis showed a HER2 score of 3 + in a needle biopsy specimen of bone metastasis. The expression of CD44v9 was slightly positive. The patient is being treated with chemotherapy with FOLFOX + trastuzumab.

**Conclusions:**

A defense mechanism against reactive oxygen species has been reported as a mechanism causing recurrence of CD44v9-positive gastric cancer. Consequently, CD44v9-positive gastric cancer grows in metastatic organs, repeatedly self-renews, and proliferates to form recurrent lesions. In the present case, the degree of CD44v9 staining in recurrent lesions was suggested to be related to the recurrence time.

## Background

Gastric cancer is the fifth most common cancer worldwide and the third most common cause of cancer-related mortality [1]. In gastric cancer, metastasis and recurrence are refractory to treatment, leading to a poor prognosis. Clinical stage, tumor size, depth of invasion, and lymph node metastasis are known prognostic factors for early recurrence; however, no independent risk factor has been identified for late recurrence at 10 years after surgery [2–4]. Local recurrence, lymph node metastases, and liver metastases occur within 3 years postoperatively, while peritoneal dissemination and other hematogenous metastases arise within 5 years postoperatively [5]. Therefore, late recurrence at 10 years after gastrectomy is extremely rare [6]. Herein, we report a para-aortic lymph node metastatic recurrence case 12 years after surgery.

## Case presentation

**Fig. 1:**
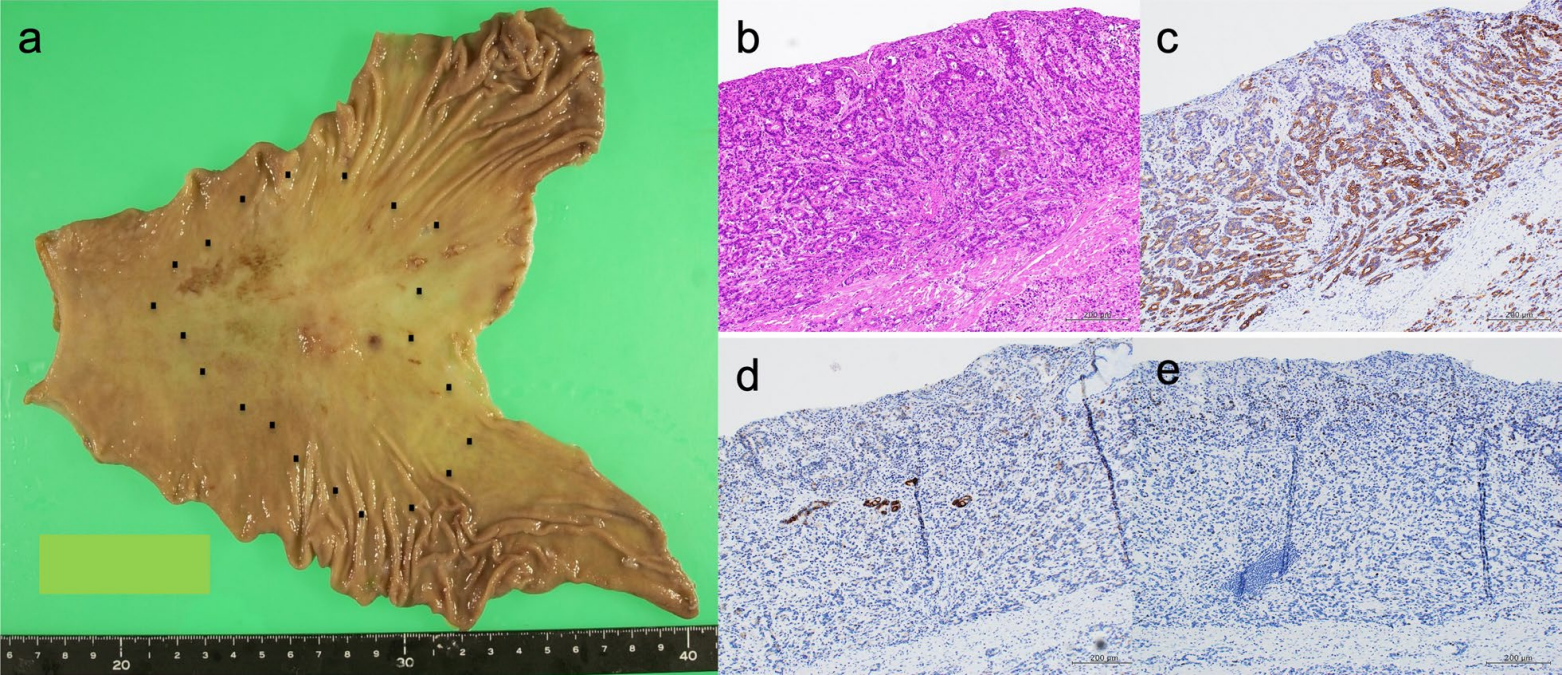
**a** The tumor presented in the lesser curvature of the gastric angle. **b** Histopathologic examination of the surgical specimen revealed the superficial extension of intermediate to poorly differentiated adenocarcinoma and partial invasion of the serous membrane. On immunohistochemical analysis, the tumor cells were noted to be positive for CK7 (**c**) and negative for CK20 (**d**) and showed a Ki-67 labeling index of < 3% (**e**)

**Fig. 2:**
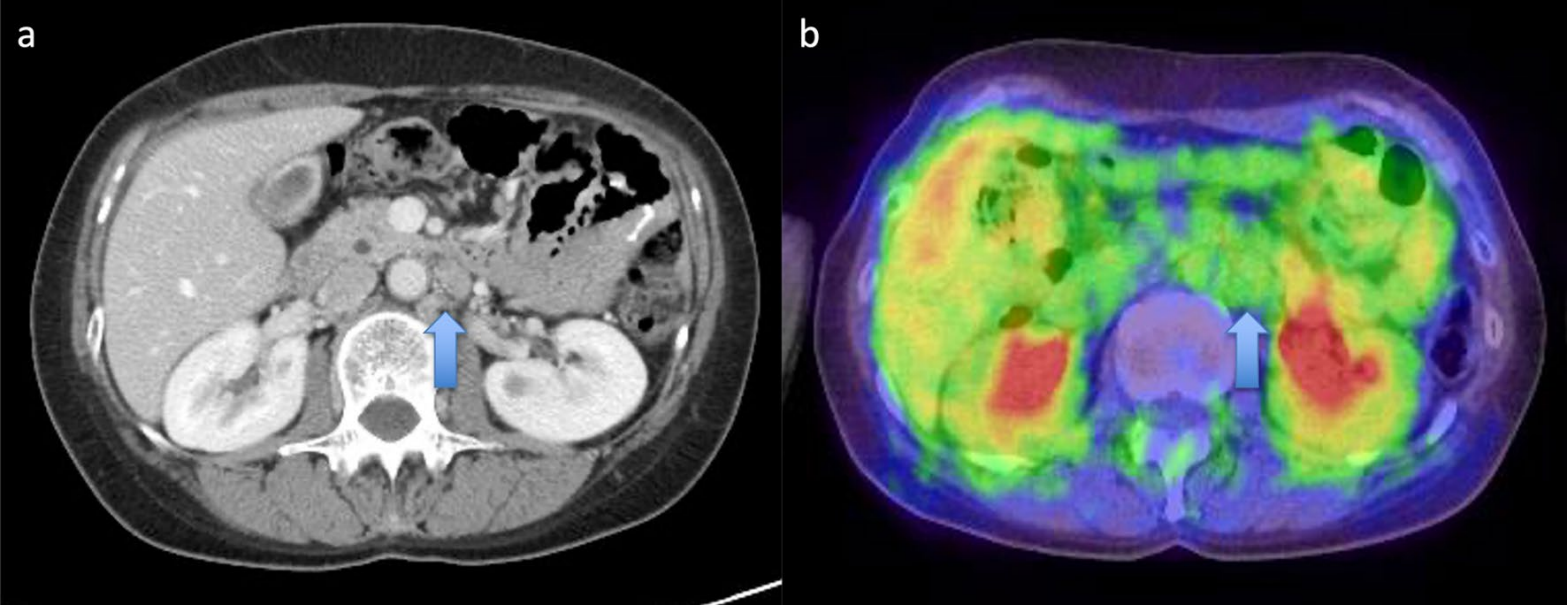
At postoperative year 5, computed tomography (**a**) /positron emission tomography (**b**) showed a 19 × 12 mm lymph node with no abnormal uptake in the No. 16b1lat lymph node station

**Fig. 3:**
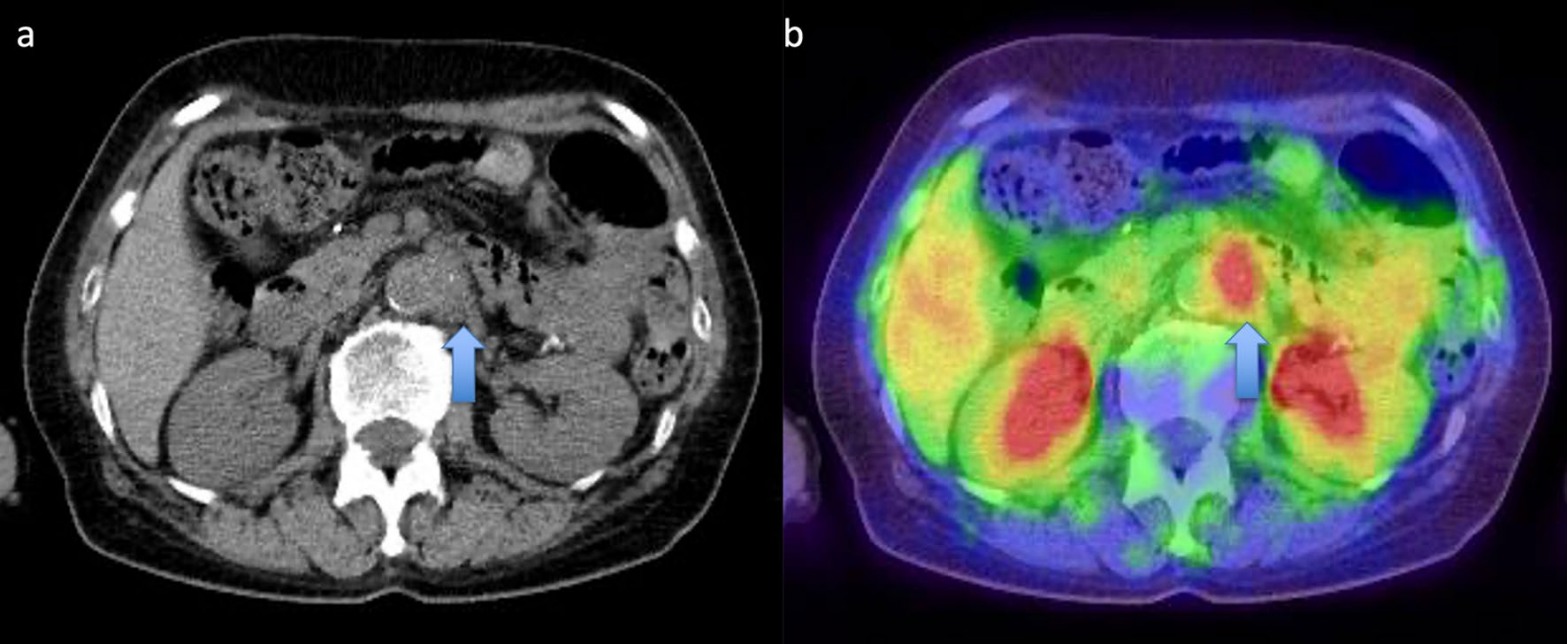
At postoperative year 12, computed tomography (**a**) / positron emission tomography (**b**) detected a 30 × 14 mm lymph node with abnormal uptake in the No. 16b1lat lymph node station

**Fig. 4:**
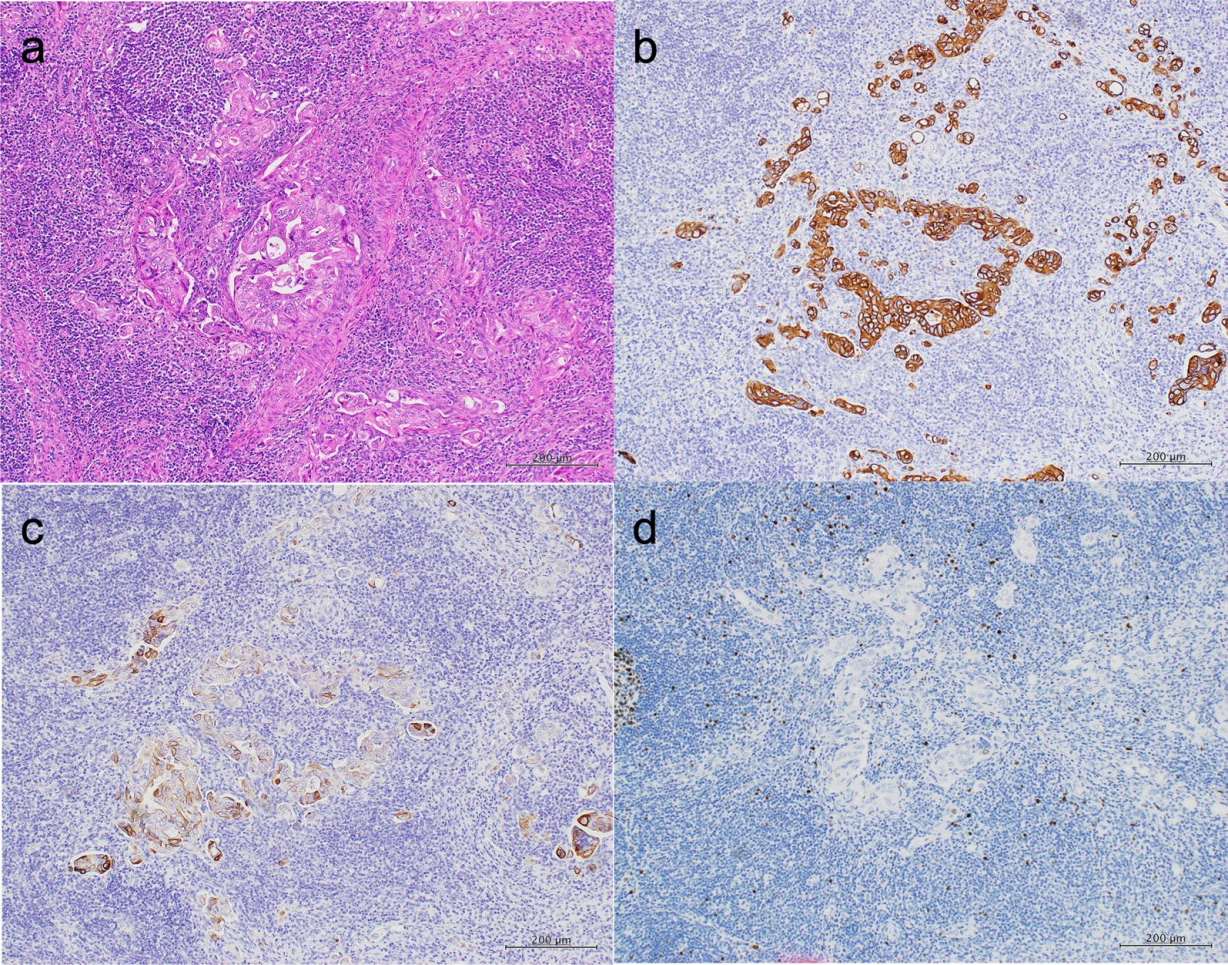
**a** Histopathologically, proliferating adenocarcinoma cells demonstrated conjoined tubular structures. On immunohistochemical analysis, the tumor cells were positive for CK7 (**b**) and negative for CK20 (**c**) and showed a Ki-67 labeling index of less than 3% (**d**)

**Fig. 5:**
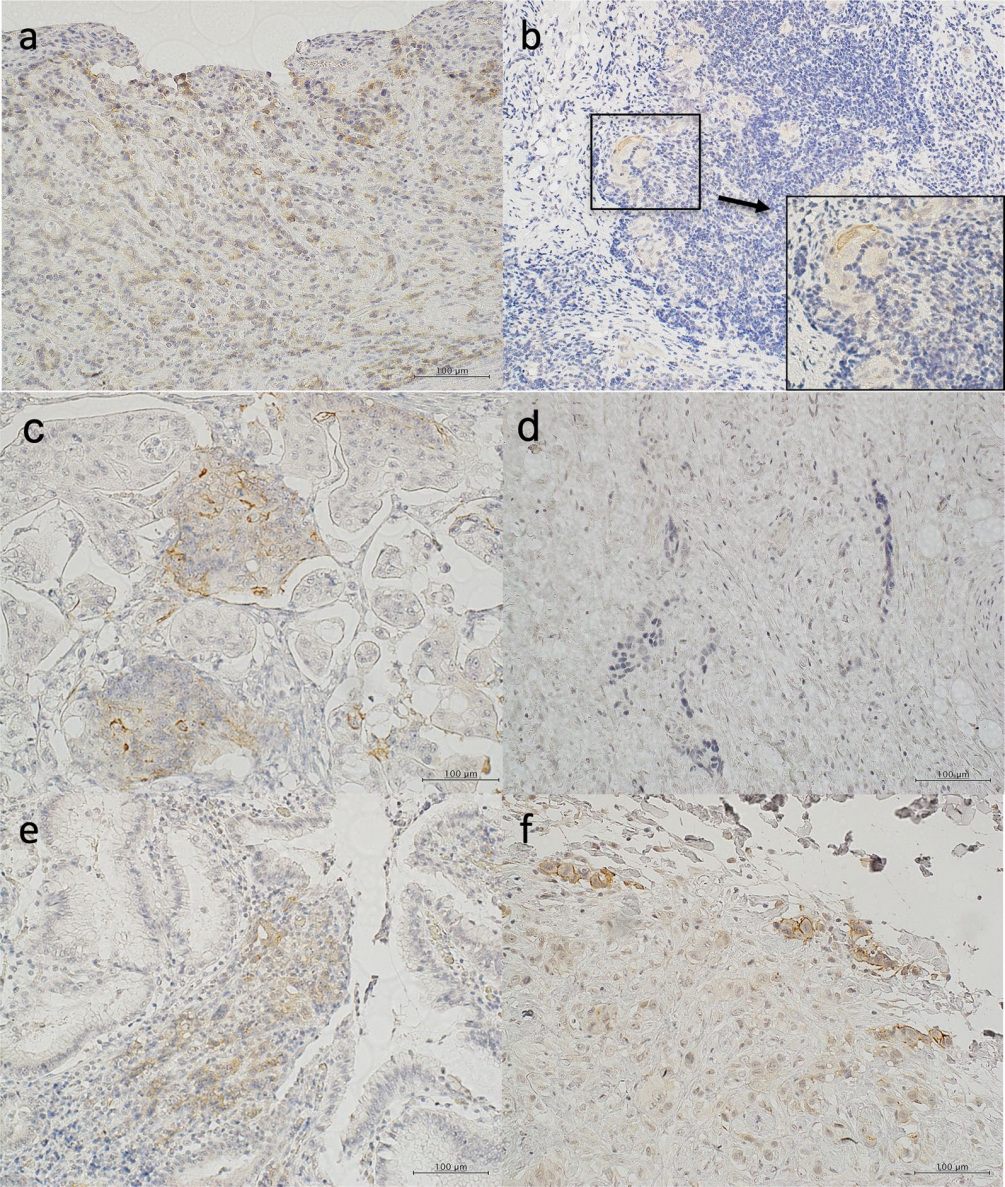
Case 1 (the present case): gastric cancer that recurred in para-aortic lymph nodes 12 years postoperatively. The expression of CD44 variant 9 (CD44v9) as primary (**a**)/recurrence (**b**): positive/slightly positive. Case 2: gastric carcinoma with para-aortic lymph node recurrence 5 years postoperatively. The expression of CD44v9 as primary (**c**)/recurrence (**d**): positive/negative or unclear. Case 3: esophagogastric junction carcinoma with skin metastasis 8 months postoperatively. The expression of CD44v9 as primary (**e**)/recurrence (**f**): positive/positive

A 44-year-old woman presented to our hospital with a 1-month history of appetite loss. A barium study and endoscopic examination revealed type 0–IIa + IIc gastric cancer with a pathologic diagnosis of moderately differentiated adenocarcinoma in the lesser curvature of the gastric angle. Contrast-enhanced computed tomography (CT) showed no lymph node swelling or distant metastasis. The serum carcinoembryonic antigen (CEA) level was 0.6 ng/mL, and the carbohydrate antigen 19-9 (CA19-9) level was 9.6 ng/mL. The preoperative diagnosis was cT1(M)cN0cM0cStageIA (Japanese Classification of Gastric Carcinoma [The 13th Edition]). She underwent laparoscopic distal gastrectomy (D1 +) + Roux-en-Y reconstruction. Histopathologic examination of the surgical specimen revealed a superficial extension of moderately to poorly differentiated adenocarcinoma and partial invasion of the serous membrane (T2:SS). Severe lymphatic invasion (ly3), moderate venous invasion (v2), and lymph node metastases in No. 1, 3, 7, and 9 lymph node stations (N2) were observed. The postoperative diagnosis was pT2(SS)pN2cM0pStageIIIA (Japanese Classification of Gastric Carcinoma [The 13th Edition]) (Fig. 1). The patient received adjuvant chemotherapy with tegafur-uracil (400 mg/day) for 2 years. Blood tests, including tumor marker tests and a whole-body CT, were performed every 3 months and 6 months, respectively. Endoscopic examination was conducted at least annually. At postoperative year (POY) 5, a 19 × 12-mm lymph node was detected in the No. 16b1lat lymph node station (Japanese Classification of Gastric Carcinoma [The 13th Edition]) (Fig. 2a). However, positron emission tomography (PET) revealed no abnormal uptake, and the levels of tumor markers were within normal limits (CEA, 1.3 ng/mL; CA19-9, 20.3 U/mL) (Fig. 2b); hence, the possibility of metastasis was considered to be low, and the patient was placed under observation. At POY 6, PET revealed no abnormal uptake. Annual whole-body CT showed no increase in tumor size, and the tumor marker expression levels remained within normal limits. At POY 10, a blood examination revealed that the CA19-9 level was elevated to 39.2 U/mL, although no increase in size was noted. At POY 12, the No. 16b1lat lymph node station was enlarged at 30 × 15 mm, the PET revealed abnormal uptake in the same lesion, and the CA19-9 level was further elevated to 72.6 U/mL (Fig. 3a, b). No other tumor was noted during esophagogastroduodenoscopy and colonoscopy. Endoscopic ultrasound-guided fine-needle aspiration revealed a moderately differentiated adenocarcinoma, similar to the previous surgery specimen’s histology. The immunohistochemistry (IHC) analysis showed that the HER2 score was negative; hence, a diagnosis of recurrence of gastric cancer was made. After two cycles of chemotherapy with tegafur–gimeracil–oteracil (120 mg/day) + oxaliplatin (100 mg/m^2^), the recurrent lesion decreased in size to 18 × 9 mm (RECIST: PR; partial response), and tumor marker levels also decreased (CEA: 1.1 ng/mL, CA19-9: 20.3 U/mL). Para-aortic nodal dissection (PAND) of No. 16b1lat and int stations was performed. In the dissected lymph nodes, proliferating adenocarcinoma cells demonstrating conjoined tubular structures were observed, a finding consistent with that of gastric cancer metastasis (Fig. 4a). Scar-like fibrosis and aggregation of foamy histiocytes were observed around the adenocarcinoma cells, indicating that a part of the tumor had undergone necrosis following chemotherapy. The immunohistochemical staining showed that CK7 was expressed in primary and recurrent specimens (Figs. 1c, 4b). Immunohistochemical staining for CK20 expression was negative in primary and recurrent specimens (Figs. 1d, 4c). The degree of staining for CD44 variant 9 (CD44v9) was attenuated in recurrent specimens compared with primary specimens (Fig. 5a, b). Based on these immunohistochemical findings and the exclusion of other malignancies on preoperative examination, gastric cancer recurrence was diagnosed. IHC test showed a HER2 score was negative in recurrent specimens. Postoperatively, the patient received chemotherapy with tegafur–gimeracil–oteracil (80 mg/day) for 1 year. At POY4, after PAND bone metastasis was confirmed in a needle biopsy specimen of bone metastasis, the pathological finding showed adenocarcinoma, and the IHC analysis showed a HER2 score of 3 + . The expression of CD44v9 was slightly positive. The patient is being treated with chemotherapy with FOLFOX + rastuzumab.

## Discussion and conclusions

We encountered a case of a late recurrence of gastric cancer at POY 12. The histopathological findings were useful in confirming the diagnosis of postoperative recurrence of gastric cancer. The histology of the recurrent tumor indicated the proliferation of adenocarcinoma cells, similar to the histology of the previous surgical specimen. Gastric adenocarcinomas demonstrate either a CK7 or CK20 expression pattern; the recurrent lesion was CK7 positive/CK20 negative, consistent with the expression pattern of the primary lesion. No epithelial tumor was detected in esophagogastroduodenoscopy and colonoscopy, and other cancers were not suspected based on contrast-enhanced CT and PET results. These findings further support the diagnosis of recurrence of gastric cancer.

To consider the reasons for late recurrence, we investigated the expression of CD44v9 with immunohistochemical staining. CD44 is a marker for cancer stem cells (CSCs) found in various solid tumors, such as breast, colon, and gastric cancers. It is a major adhesion molecule in the extracellular matrix. [7] CSCs, a unique subpopulation in tumors, can initiate tumor growth and sustain tumor self-renewal [8]. CD44 gene transcripts undergo complex alternative splicing, among which CD44v9 expression is significantly associated with poor prognosis concerning overall survival and recurrence-free survival [9–13]. CD44v9 stabilizes the glutamate–cystine transporter and promotes the uptake of cystine, which is required for glutathione (GSH) synthesis in the cell [14]. GSH is cells’ most abundant non-enzymatic antioxidant molecule and directly removes intracellular reactive oxygen species (ROS) [14]. GSH peroxidase 2, the gastrointestinal form of GSH peroxidase, is an antioxidant enzyme that catalyzes the reduction of intracellular ROS using GSH as a reductant. This defense mechanism against ROS prevents apoptosis of CSCs, making them resistant to anticancer drug therapy and radiotherapy [15]. Conversely, silencing CD44v9 with siRNA has been reported to promote apoptosis and cell cycle arrest and inhibit cell proliferation [16]. In our case, the expression of CD44v9 was attenuated in the recurrent lesion compared to the primary lesion (Fig. 5a, b). The defense mechanism against ROS in the recurrent lesion may be weakened by the decreased expression of CD44v9. When the number of CD44v9-positive cells is low, tumor formation and cell proliferation may also be suppressed. The competing activities of cell proliferation and apoptosis may contribute to tumor growth inhibition. Meanwhile, the Ki-67 labeling index of < 3% (Figs. 1e, 4d) and the weak accumulation of fluorodeoxyglucose by PET suggest that the tumor cells are in a state of cell cycle arrest or dormancy [17]. Therefore, the expression of CD44v9 in recurrent lesions may cause late recurrence.

We hypothesized that the degree of CD44v9 expression in recurrent lesions correlates with the recurrence time. Existing reports suggested that CD44v9-positive tumors have a higher recurrence rate and poor survival than CD44v9-negative tumors [9–13]. On the other hand, no papers have examined the expression of CD44v9 in recurrent tumors. We investigated the expression of CD44v9 in the primary and recurrent lesions in seven patients who underwent radical gastric cancer surgery and resection of recurrent lesions in our hospital. Among them, CD44v9 expression was positive in three cases. Case 1 was the present case of gastric cancer with para-aortic lymph node recurrence 12 years postoperatively, Case 2 was gastric carcinoma with para-aortic lymph node recurrence 5 years postoperatively, and Case 3 was esophagogastric junction carcinoma with skin metastasis 8 months postoperatively. The expression of CD44v9 was classified as primary/recurrence: positive/slightly positive in Case 1, positive/negative or unclear in Case 2, and positive/positive in Case 3. In Case 3 (early recurrence), high expression of CD44v9 in the recurrent lesion was observed. We suspect that in gastric cancers expressing CD44v9, cases with a lower percentage of CD44v9 expression in the recurrent lesion have a longer time to recurrence than those with a higher percentage of CD44v9 expression.

A limitation of our study is that this conclusion is drawn from a small number of cases. There are only a few cases in which surgery for gastric cancer recurrence is indicated, and consequently, pathological findings in recurrent lesions are rarely reviewed.

A defense mechanism against ROS has been reported as a mechanism that causes the recurrence of CD44v9-positive gastric cancer. Consequently, CD44v9-positive gastric cancer grows in metastatic organs, repeatedly self-renews, and proliferates to form recurrent lesions. In the present case, the degree of CD44v9 staining in recurrent lesions was suggested to be related to the recurrence time.

## Data Availability

The data can be accessed from PubMed (https://pubmed.ncbi.nlm.nih.gov) or the Japan Medical Abstracts Society (https://search.jamas.or.jp).

## Abbreviations

CA19-19: Carbohydrate antigen 19-9
CD44v9: CD44 variant 9
CEA: Carcinoembryonic antigen
CSCs: Cancer stem cells
CT: Computed tomography
GSH: Glutathione
PAND: Para-aortic nodal dissection
PET: Positron emission tomography
POY: Postoperative year
PR: Partial response
ROS: Reactive oxygen species
IHC: Immunohistochemistry

## References

[1] Ferlay J, Soerjomataram I, Dikshit R, Eser S, Mathers C, Rebelo M (2015). Cancer incidence and mortality worldwide: sources, methods and major patterns in GLOBOCAN 2012. Int J Cancer.

[2] Shiraishi N, Inomata M, Osawa N, Yasuda K, Adachi Y, Kitano S (2000). Early and late recurrence after gastrectomy for gastric carcinoma. Univariate and multivariate analyses Cancer.

[3] Lee JH, Kim HI, Kim MG, Ha TK, Jung MS, Kwon SJ (2016). Recurrence of gastric cancer in patients who are disease-free for more than 5 years after primary resection. Surgery.

[4] Moon YW, Jeung HC, Rha SY, Yoo NC, Roh JK, Noh SH (2007). Changing patterns of prognosticators during 15-year follow-up of advanced gastric cancer after radical gastrectomy and adjuvant chemotherapy: a 15-year follow-up study at a single Korean institute. Ann Surg Oncol.

[5] Takahashi R, Ohashi M, Kano Y, Ida S, Kumagai K, Nunobe S (2019). Timing and site-specific trends of recurrence in patients with pathological stage II or III gastric cancer after curative gastrectomy followed by adjuvant S-1 monotherapy. Gastric Cancer.

[6] Ogasawara N, Ohkura Y, Ueno M, Haruta S, Nakayama A, Fujii T (2020). Late recurrence of gastric carcinoma 15 years after surgery. Clin J Gastroenterol.

[7] Takaishi S, Okumura T, Tu S, Wang SS, Shibata W, Vigneshwaran R (2009). Identification of gastric cancer stem cells using the cell surface marker CD44. Stem Cells.

[8] Lau WM, Teng E, Chong HS, Lopez KA, Tay AY, Salto-Tellez M (2014). CD44v8-10 is a cancer-specific marker for gastric cancer stem cells. Cancer Res.

[9] Jogo T, Oki E, Nakanishi R, Ando K, Nakashima Y, Kimura Y (2021). Expression of CD44 variant 9 induces chemoresistance of gastric cancer by controlling intracellular reactive oxygen spices accumulation. Gastric Cancer.

[10] Yasui W, Kudo Y, Naka K, Fujimoto J, Ue T, Yokozaki H (1998). Expression of CD44 containing variant exon 9 (CD44v9) in gastric adenomas and adenocarcinomas: relation to the proliferation and progression. Int J Oncol.

[11] Hirata K, Suzuki H, Imaeda H, Matsuzaki J, Tsugawa H, Nagano O (2013). CD44 variant 9 expression in primary early gastric cancer as a predictive marker for recurrence. Br J Cancer.

[12] Go SI, Ko GH, Lee WS, Kim RB, Lee JH, Jeong SH (2016). CD44 variant 9 serves as a poor prognostic marker in early gastric cancer, but not in advanced gastric cancer. Cancer Res Treat.

[13] Yamakawa Y, Kusuhara M, Terashima M, Kinugasa Y, Sugino T, Abe M (2017). CD44 variant 9 expression as a predictor for gastric cancer recurrence: immunohistochemical and metabolomic analysis of surgically resected tissues. Biomed Res.

[14] Ishimoto T, Nagano O, Yae T, Tamada M, Motohara T, Oshima H (2011). CD44 variant regulates redox status in cancer cells by stabilizing the xCT subunit of system xc(−) and thereby promotes tumor growth. Cancer Cell.

[15] Miyoshi S, Tsugawa H, Matsuzaki J, Hirata K, Mori H, Saya H (2018). Inhibiting xCT improves 5-fluorouracil resistance of gastric cancer induced by CD44 variant 9 expression. Anticancer Res.

[16] Suwannakul N, Ma N, Midorikawa K, Oikawa S, Kobayashi H, He F (2020). CD44v9 induces stem cell-like phenotypes in human cholangiocarcinoma. Front Cell Dev Biol.

[17] Schewe DM, Aguirre-Ghiso JA (2008). ATF6alpha-Rheb-mTOR signaling promotes survival of dormant tumor cells in vivo. Proc Natl Acad Sci USA.

